# Glucocorticoids regulate pentraxin-3 expression in human airway smooth muscle cells

**DOI:** 10.1371/journal.pone.0220772

**Published:** 2019-08-22

**Authors:** Jingbo Zhang, Latifa Koussih, Lianyu Shan, Andrew J. Halayko, Omar Tliba, Abdelilah S. Gounni

**Affiliations:** 1 Department of Immunology, Max Rady College of Medicine, Rady Faculty of Health Sciences, University of Manitoba, Winnipeg, Manitoba, Canada; 2 Department of Experimental Sciences, University of Saint Boniface, Winnipeg, Manitoba, Canada; 3 Department of Physiology and Pathophysiology, University of Manitoba, Max Rady College of Medicine, Rady Faculty of Health Sciences, Winnipeg, Manitoba, Canada; 4 Department of Biomedical Sciences, College of Veterinary Medicine, Long Island University, Brookville, New York, United States of America; University of PECS Medical School, HUNGARY

## Abstract

Pentraxin-3 (PTX3) is a multifunctional protein involved in both innate and adaptive immunity. Glucocorticoid (GC) is the first-line therapy to mitigate airway inflammation in asthma. Previous pieces of evidence showed that GC has divergent effects on PTX3 production in various cell types. The molecular mechanisms controlling PTX3 expression in HASMC are, however, not yet characterized. In this study, we demonstrate that the synthetic GC, dexamethasone (DEX) increases the expression of PTX3 both at the protein and mRNA levels. We also found that such an effect of DEX was dependent on *de novo* protein synthesis and the GC receptor (GR). While DEX increases PTX3 mRNA stability, it did not affect its promoter activity. Interestingly, HASMC pre-treated with p42/p44 ERK inhibitor, but not with p38 or JNK-MAPK inhibitors, significantly interfered with DEX-induced PTX3 secretion. Taken together, our data suggest that GC regulates PTX3 expression in HASMC through transcriptional and post-transcriptional mechanisms in a GR and ERK-dependent manner.

## Introduction

Asthma is characterized by recurrent episodes of airflow obstruction and airway hyper-responsiveness (AHR). These symptoms are the result of chronic airway remodeling featured by smooth muscle outgrowth, mucus hypersecretion, and inflammation.[1] During asthma, the airways are continuously exposed to environmental allergens and undergo a constant cycle of damage and repair, causing epithelial distortion, goblet cell hyperplasia [2], and the alteration of the underlying mesenchymal layers resulting in airway smooth muscle (ASM) hyperplasia and hypertrophy of and subepithelial fibrosis.[3, 4] These structural changes represent the major contributing factors to airway narrowing, airflow obstruction, and AHR. At present, however, the pathogenic mechanisms controlling airway remodeling remain poorly understood.

Long pentraxin 3 (PTX3) is a soluble pattern recognition receptor produced by a variety of cells at the site of infection or inflammation [5, 6]. As a component of the humoral arm of innate immunity, PTX3 is a multifunctional protein involved in the defense against various pathogens, in the assembly of a hyaluronic acid-rich extracellular matrix, and female fertility, angiogenesis, and cardiovascular diseases. PTX3 production is commonly induced by primary inflammatory signals (e.g., TLR engagement, TNF, and IL-1β) [5].

Glucocorticoids (GC) are first-line therapy to mitigate airway inflammation in asthma. Recent evidence showed that GCs were able to modulate PTX expression. However, such effects were variable and highly cell-specific. Indeed, while GCs inhibits the PTX3 secretion in myeloid dendritic cells (DC), in fibroblasts and endothelial cells (EC), GC enhances PTX3 secretion [7]. The mechanisms underlying such divergent effects of GC on PTX3 secretion remain, however, yet to be investigated [8].

Previously, we showed that PTX3 expression is increased in bronchial biopsies and bronchoalveolar lavage fluid of subjects with severe asthma when compared to healthy donors [9]. More recently, we demonstrated that *PTX3* deficient mice exhibit enhanced inflammation, AHR and mucus production upon OVA sensitization and challenge [10] strongly suggesting a protective role of PTX3 in a murine model of allergic asthma.

ASM cells are a prominent cellular target for inhaled GC [11]. However, the role of GC in regulating PTX3 expression by HASMC remains unknown. In the current study, we showed in HASMC, that dexamethasone (DEX) whether alone or in combination with TNF increases PTX3 mRNA expression and protein secretion, and this in a GC receptor (GR)-dependent manner. Inhibitor of transcription actinomycin D (ActD) abolishes the effect of DEX either alone or in combination with TNF on PTX3 mRNA expression. Transfection studies using proximal PTX3 promoter luciferase reporter showed that DEX-induced PTX3-dependent gene transcription. Intriguingly, we also found that while DEX was able to enhance TNF-induced PTX3 secretion, it significantly inhibited TNF-induced PTX3 promoter activity. Finally, we showed in cells treated with DEX either alone or in combination with TNF that ERK1/2 MAPK inhibition, but not JNK or p38, significantly decreased PTX3 protein secretion. Our data suggest that GC regulates PTX3 expression in HASMC through transcriptional and post-transcriptional mechanisms in a GR and ERK-dependent manner.

## Materials and methods

### Reagents

DEX was purchased from Sigma (St. Louis, MO). Recombinant human TNF, PTX3, mouse anti-human ERK1/2 mAb, affinity purified rabbit anti-phosphoERK1/2 (T202/Y 204) and ELISA kits for human PTX3 were purchased from R&D Systems (Minneapolis, MN). The p38 MAPK inhibitor, SB-203580[4-4-fluorophenyl)-2-(4-methyl-sulfinylphenyl)-5- (4’-pyridyl)-1H-imidazole], the p42/ p44 ERK inhibitor, U-0126 [1, 4-diamino-2, 3-dicyano-1, 4-bis (2-aminophenyl-thio) butadiene], and the PI3K inhibitor, wortmannin, were purchased from Calbiochem (Mississauga, Ontario, Canada). All cell culture media (DMEM and F-12), antibiotics (penicillin, streptomycin), trypsin-EDTA, and cell culture reagents were obtained from Invitrogen Life Technologies, and FBS was from HyClone Laboratories (Logan, UT). The anti-human smooth muscle actin antibody (Ab) was obtained from DakoCytomation. Alkaline phosphatase-conjugated streptavidin was purchased from Jackson ImmunoResearch Laboratories. Unless stated otherwise, all other reagents were obtained from Sigma-Aldrich.

### Preparation and culture of HASMC

HASMC were obtained from macroscopically healthy segments after lung resection from surgical patients following the procedures approved by the Human Research Ethics Board of the University of Manitoba, Winnipeg, Canada. Written informed consent was obtained from each. Primary HASMC were isolated from explants, as described previously [12].

At confluence, primary HASMC exhibited spindle morphology and a hill-and-valley pattern that is characteristic of smooth muscle in culture. HASMC were grown in DMEM supplemented with 10% FBS and antibiotics (100 U/ml penicillin and 100 μg/ml streptomycin). Moreover, using culture up to passage 5, over 90% of the cells at confluence retain smooth muscle-specific actin, SM22, calponin protein expression and mobilize intracellular Ca^2+^ in response to acetylcholine, a physiologically relevant contractile agonist. In all experiments, cells were used at passages 3–5.

### Cell stimulation and ELISA assay

Confluent HASMC were growth arrested by FBS deprivation for 48 h in Ham’s F-12 media containing 5μg/ml human recombinant insulin, 5μg/ml human transferrin, 5ng/ml selenium, and antibiotics (100 U/ml penicillin and 100μg/ml streptomycin). Cells were then stimulated in fresh FBS-free media with a graded concentration of DEX (10^−7^ M, 10^−6^ M, and 10^−5^ M), TNF (10ng/ml) or in combination. DEX was used at 10^−5^ M and 10^−6^ M concentrations, concentrations previously shown to abrogate TNF-induced the secretion of several cytokines in ASM cells such as IL-6, RANTES and eotaxin1/CCL11 [13, 14].

In some experiments, cells were pretreated for 30 min with RU486 (10 μM), U-0126 (10μM), SB-203580 (10μM), SP-600125 (50nM), actinomycin D (Act D) (5μg/ml) before DEX (10^−6^ M) and TNF (10 ng/ml), was added for 24 h.

Supernatants were then collected and analyzed for PTX3 using ELISA according to the manufacturer’s protocol (R&D Systems).

Trypan blue assay and propidium iodide staining combined with flow cytometry showed no statistical difference between Act D (1, 5 or 10μg/ml) treated and untreated ASM cells (S1 Fig).

### RNA isolation and real-time RT-PCR analysis

Total cellular RNA was extracted using Trizol (Gibco BRL; Gaithersburg; MD). Reverse transcription was performed as we described previously. [9]. Relative levels of PTX3 mRNA were assessed by quantitative real-time RT-PCR analysis using the SYBR Green (SYBR Premix Ex Taq RT-PCR kit; Takara, Shiga, Japan), Light-Cycler (Roche). The sequences of primers were as follow: PTX3 forward, 5′-GGGACAAGCTCTTCATCATGCT-3′; reverse, 5′-GTCGTCCGTGGCTTGCA-3′; glyceraldehyde-3-phosphate dehydrogenase (GAPDH) served as the internal control. Primers for the house-keeping gene (GAPDH) are as follows: forward primer 5'-AGCAATGCCTCCTGCACCACCAAC-3', and reverse primer 5'-CCGGAGGGGCCATCCA. Total 40 cycles were run with each cycle including the following steps: denaturation (94°C, 1 min), annealing (62°C, 32s) and extension (72°C, 1 min 32 s).

### PTX3 mRNA stability

Growth-arrested HASMC were stimulated with TNF (10 ng) alone or in combination with DEX (10^−6^ M) for 10 h before the addition of 5 μg/ml Act D. Total cellular RNA was then extracted at the indicated time points post-Act D incubation, and mRNA expression was quantified by real time-RT-PCR. PTX3 mRNA copy numbers were normalized to the respective GAPDH values. Results are presented as the % mRNA remaining compared with the initial 9 h culture time point. One phase exponential decay constant (*k*) were calculated by nonlinear regression of the % mRNA remaining vs. time of Act D treatment using GraphPad Prism software (v.4.0) as we previously described [15]

### PTX3 promoter luciferase reporter constructs and cell transfection

The luciferase reporter plasmid harboring the human PTX3 promoter was kindly provided by Dr. Andrea Doni [7]. Transfection was performed with ExGen 500 according to the manufacturer’s instructions (Fermentas Inc, Mississauga, ON). Luciferase activity was measured as we previously described [16]. Briefly, HASMC (3.5×10^4^) were seeded into 24-well plates in complete media (DMEM/10% FBS). At 70% confluency, transfection was performed in triplicate using ExGen 500 according to the manufacturer’s instructions (Fermentas, Mississauga, ON). In each well, 0.8 μg of PTX3 promoter-luciferase DNA construct was added and Renilla luciferase reporter vector (pRL-TK 0.2 μg/sample) was co-transfected and incubated for 24 h. The media were then changed, and cells were stimulated with DEX (10^−6^ M) and/or TNF (10ng/ml) for 12h [16]. Luciferase activity was measured using the Dual-Luciferase Reporter Assay System (Promega) and a luminometer (LB9501, Berthold Lumat). Briefly, 20 μl of cell lysate was mixed with 100 μl of Luciferase Assay Reagent II, and firefly luciferase activity was recorded. One-hundred microliters of Stop and Glo Reagent (Promega) was then added, and Renilla luciferase activity was again measured. All values were normalized to the mock Renilla luciferase activity.

### Western blot analysis

Nearly confluent ASM cells were growth arrested by FBS deprivation for 48h as described above. Cells were then stimulated in fresh FBS free medium with DEX, TNF (10 ng/ml), combination or medium alone. At selected time points, the cells were washed once with cold phosphate buffered saline (PBS), and total proteins were extracted with lysis buffer (1% NP-40, PMSF, 2mM sodium vanadate, 0.1% sodium deoxycolate, and protease inhibitor cocktail (Hoffmann-LaRoche Limited, Mississauga, ON). Harvested lysates were centrifuged for 10 min at 4 °C to pellet cellular debris. The supernatants were removed and stored at -70°C. Protein lysate (10μg) were loaded on 10% SDS PAGE, followed by transfer to nitrocellulose membranes (Invitrogen). The blots were then blocked with 5% non-fat dry milk in TBS/0.1% Tween (TBST) for 1h at room temperature, and then incubated overnight at 4°C with antibodies specific for phosphorylated ERK1/2 (T202/Y204). After washing with TBST, the blots were incubated with goat anti-mouse or goat anti rabbit horseradish peroxidase conjugated secondary antibodies and bands were revealed with ECL reagents (Amersham Pharmacia, Baie D’Urfe, Quebec, Canada). After stripping, total anti-ERK was used as loading control. Densitometric analysis was performed by using Image Lab 4.1 software (Bio-Rad Laboratories, Mississauga, ON, Canada) and integrated density value was presented as the fold-increase in phosphorylated over total forms of ERK1/2.

### Statistical analyses

Data obtained from experiments performed in triplicate and was represented as means ± SEM. Differences among groups were analyzed using unpaired t-tests or ANOVA together with a post-hoc Bonferroni analysis. Non-parametric data were analyzed using the Kruskal-Wallis test followed by the Mann-Whitney U-test. P values were considered significant at 0.05 levels.

## Results

### DEX induces PTX3 protein secretion from HASMC

**Fig 1:**
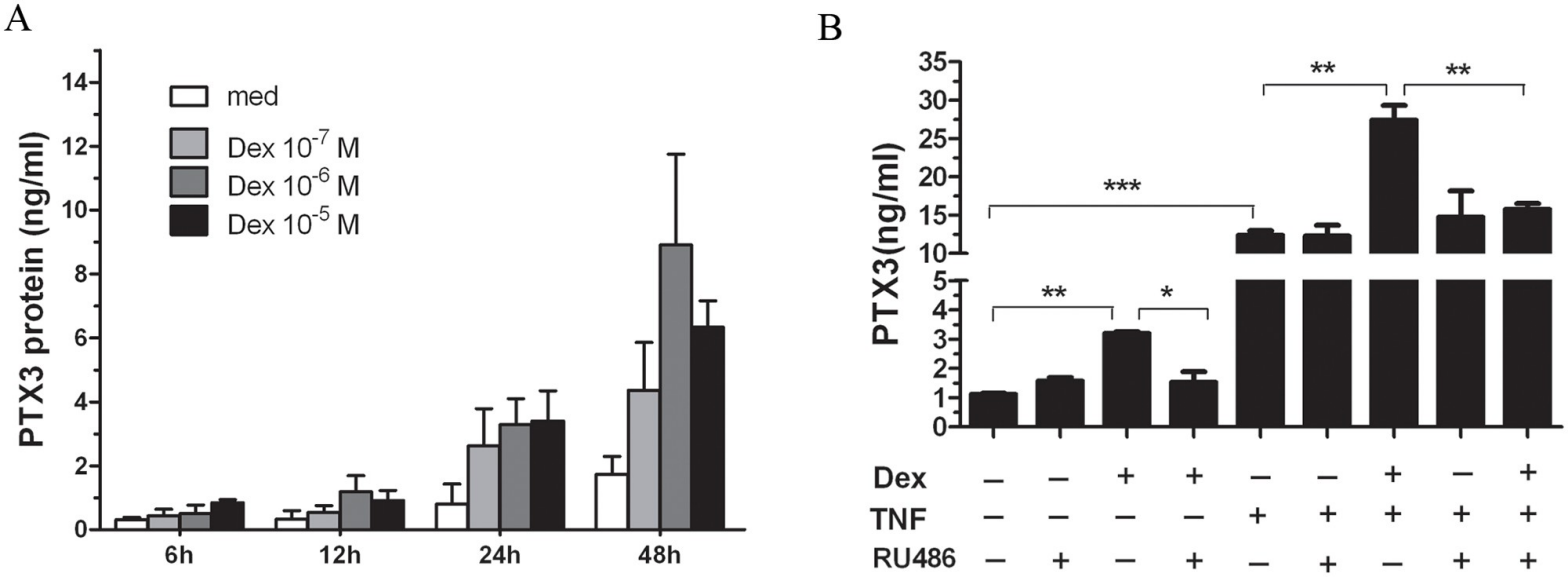
Effect of dexamethasone (DEX) and TNF on PTX3 secretion in HASMC. (A) Supernatants from serum-deprived HASMC stimulated with various concentrations of DEX (10^−7^ M, 10^−6^ M, and 10^−5^ M) were harvested at different time point (6, 12, 24 and 48 h) and analyzed by ELISA for PTX3; (B) Grow-arrested HASMC were treated with DEX (10^−6^ M) and/or TNF (10ng/ml) for 24 h and RU486 (10 μM) added 30 min before. PTX3 secretion was assessed by ELISA. Data are presented as the mean ± SD of five independent experiments in triplicate (*p<0.05, **P <0.01, ***P <0.001).

We first examined whether DEX induces PTX3 secretion in HASMC. Primary HASMC were treated with vehicle (medium alone) or various concentrations of DEX (10^−7^ M, 10^−6^ M, and 10^−5^ M) before supernatants were harvested 6, 12, 24 and 48 h later and analyzed by ELISA. A constitutive/spontaneous secretion of PTX3 was detected in vehicle-treated HASMC at 6, 12 and 24 h time points which reached a maximum at 48 h. DEX significantly increased PTX3 secretion with an optimal time and dose-response at 48 h and 10^−6^ M, respectively (Fig 1A).

Since we previously showed that TNF induces PTX3 secretion in HASMC [17], we next investigated whether DEX and TNF synergize to modulate PTX3 secretion. As shown in Fig 1B, a synergistic effect between DEX and TNF was observed resulting in a 5.05 ± 1.99-fold or a 1.91 ± 1.608-fold increase when compared to cells treated with DEX or TNF alone (Fig 1B), respectively.

We next examined whether DEX-induced PTX3 secretion was GR-dependent. To this end, HASMC were pre-treated for 30 minutes with the GR antagonist, RU486 (10μM), before DEX was added for 48 h. We found that RU486 completely abolished the DEX-induced PTX3 secretion. Moreover, the synergistic effect of DEX and TNF on PTX3 secretion was also inhibited by the addition of RU486. Of note, RU486 did not induce PTX3 nor alter the TNF-induced PTX3 production by HASMC (Fig 1B). Collectively, these data indicate that DEX-induced PTX3 specifically in HASMCs requires GR.

### DEX enhances PTX3 mRNA expression in HASMC

**Fig 2:**
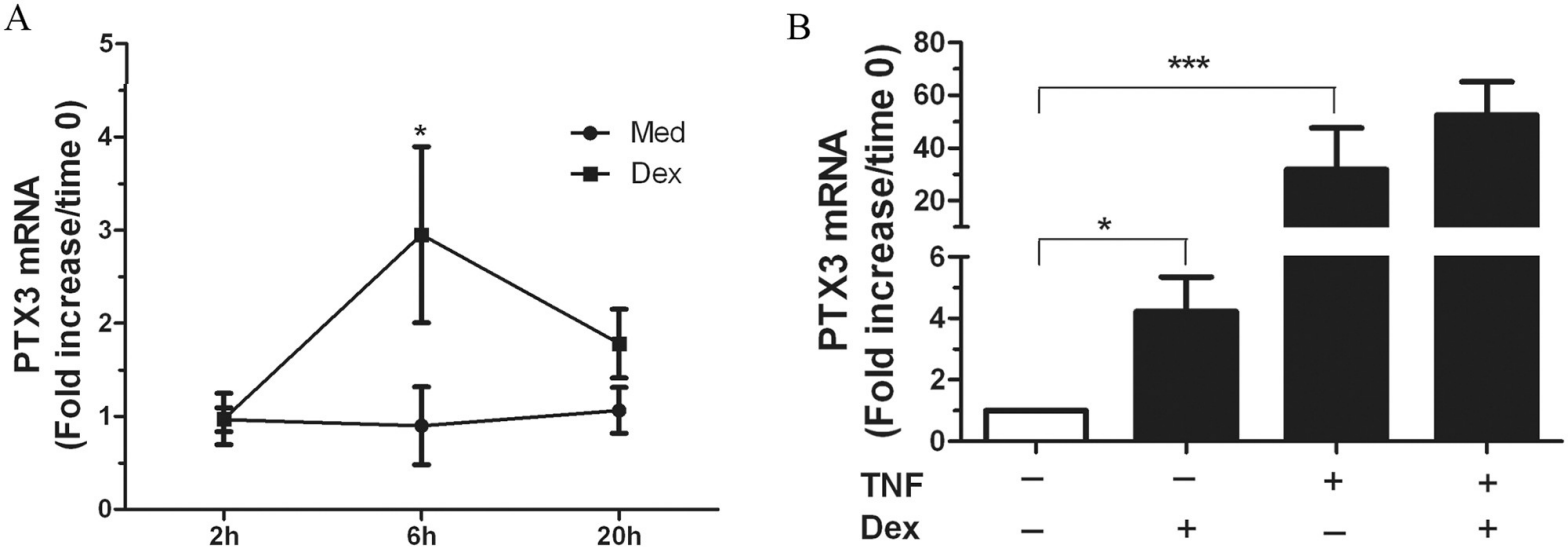
DEX enhances PTX3 mRNA expression in HASMC. Growth-arrested HASMC were treated with DEX alone (10^−6^ M) for 2, 6, and 20 h (A) or in combination with TNF (10 ng/ml) for 6 h (B). mRNA was then analyzed by real time-PCR as described in MATERIALS AND METHODS. Data are from three primary HASMC preparations, normalized with internal control GAPDH and expressed as a ratio to the time 0 baseline level (*P<0.05, *** P<0.001).

To investigate whether DEX alone or in combination with TNF affects PTX3 mRNA expression, we next examined PTX3 mRNA expression using quantitative real-time- RT-PCR. Primary HASMC were treated with DEX (10^−6^ M) or vehicle; mRNA was harvested after 2, 6, and 20 h. We found that DEX treatment significantly increases PTX3 mRNA expression reaching a maximum level at 6 h and decreases at 20 h (Fig 2A). When used separately, DEX or TNF significantly enhances PTX3 mRNA expression by a 5.102 ± 1.7 or 16.1 ± 6.1-fold increase of PTX3 mRNA when compared with cells treated with medium alone, respectively. When TNF and DEX were combined, no significant differences were observed (Fig 2B).

### DEX-induced PTX3 secretion in HASMC is dependent on de novo mRNA synthesis

**Fig 3:**
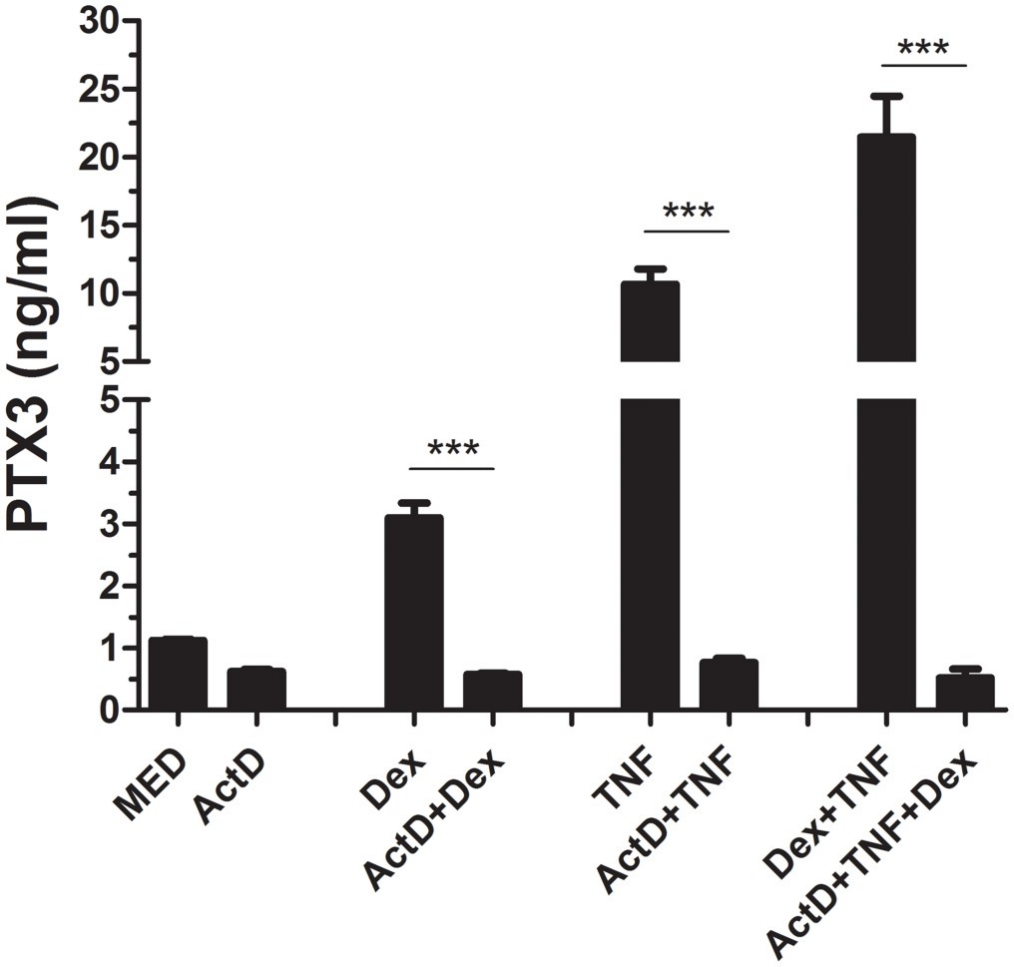
Induction of PTX3 protein release by DEX alone, or in combination with TNF depends on mRNA neosynthesis in HASMC. Serum-deprived HASMC were pre-treated with Act D for 30 min before DEX (10^−6^ M) and TNF (10 ng/ml) were added either alone or in combination for 24 h. Supernatants were then harvested analyzed by ELISA. Results are representative of 3 donors in triplicate (***P<0.001).

To examine whether the effect of DEX on PTX3 secretion depends on mRNA neosynthesis, confluent serum-deprived HASMC were pretreated with Act D (5μg/ml) for 30min then treated with DEX (10^−6^ M) and TNF (10 ng/ml) either alone or in combination for 24 h. Compared with untreated cells, pretreatment with Act D completely abrogated the induction of PTX3 secretion (*p* <0.0001) irrespective of cytokine or GC treatments (Fig 3). Together, these results suggest that the induction of PTX3 secretion in HASMC by GC or TNF cytokine depends on *de novo* mRNA synthesis.

### DEX does not affect PTX3 promoter activity

**Fig 4:**
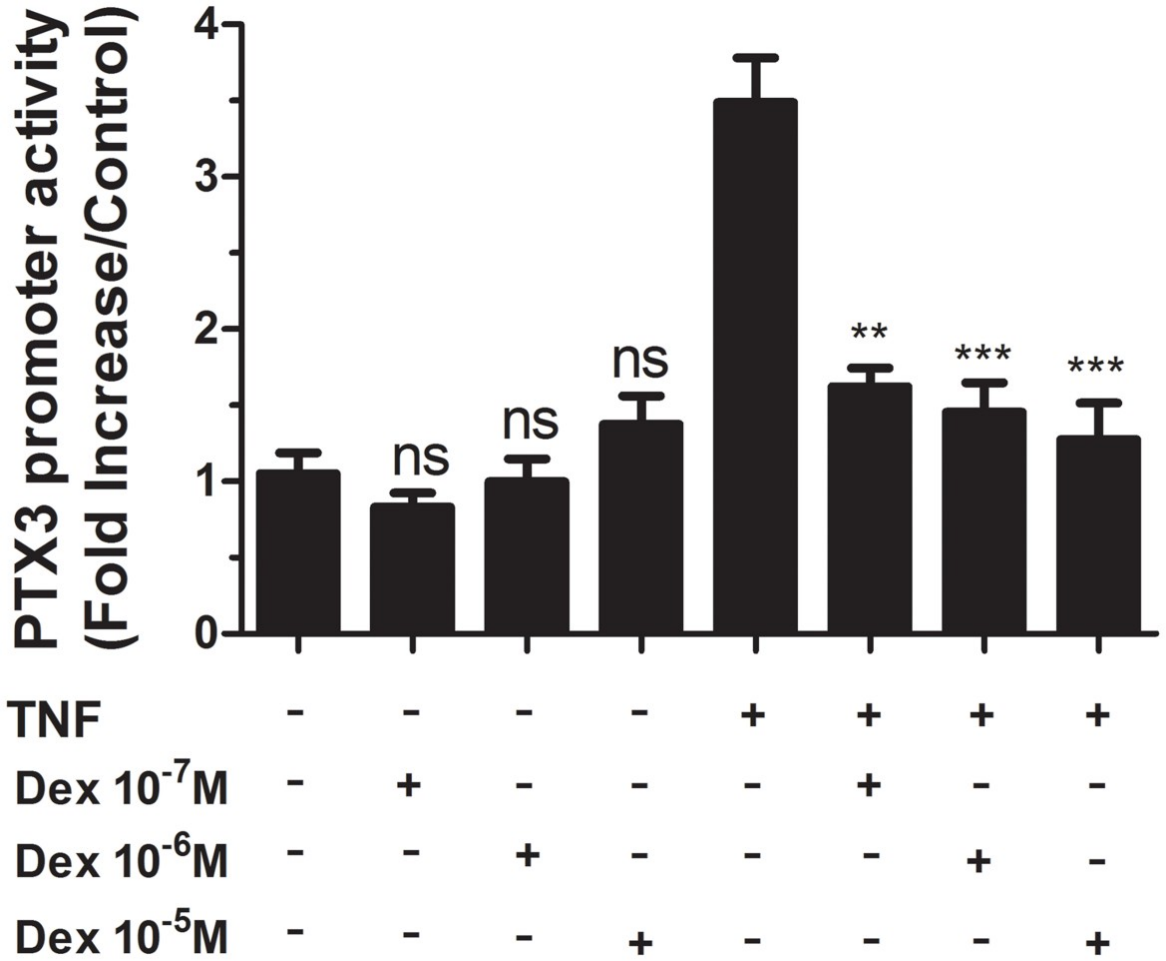
Transcriptional regulation of the PTX3 promoter by DEX. HASMC were transiently transfected with the PTX3 promoter-luciferase construct and stimulated with different concentrations of DEX either alone (10^−7^ M, 10^−6^ M, and 10^−5^ M), or with TNF (10ng/ml) for 16 h. Values are presented as mean fold increase of luciferase activity normalized to the mock Renilla luciferase activity from 4 different donors. **P<0.01,***P<0.001 compared with TNF-stimulation group; ns, not significant compared with the medium group.

To further investigate mechanisms by which DEX enhances PTX3 transcription, a reporter plasmid (pGV-B2) carrying 1.1Kb of the human PTX3 proximal promoter linked to luciferase gene [7] was used to transfect HASMC transiently. We found that PTX3 promoter activity was not affected by DEX treatment (10^-5^M) (n = 4). Strikingly, in combination, DEX inhibited significantly TNF-induced PTX3 promoter (n = 8, Fig 4). This result suggests that DEX increases TNF-induced PTX3 secretion by involving post-transcriptional mechanisms.

## Effect of DEX on PTX3 mRNA stability

**Fig 5:**
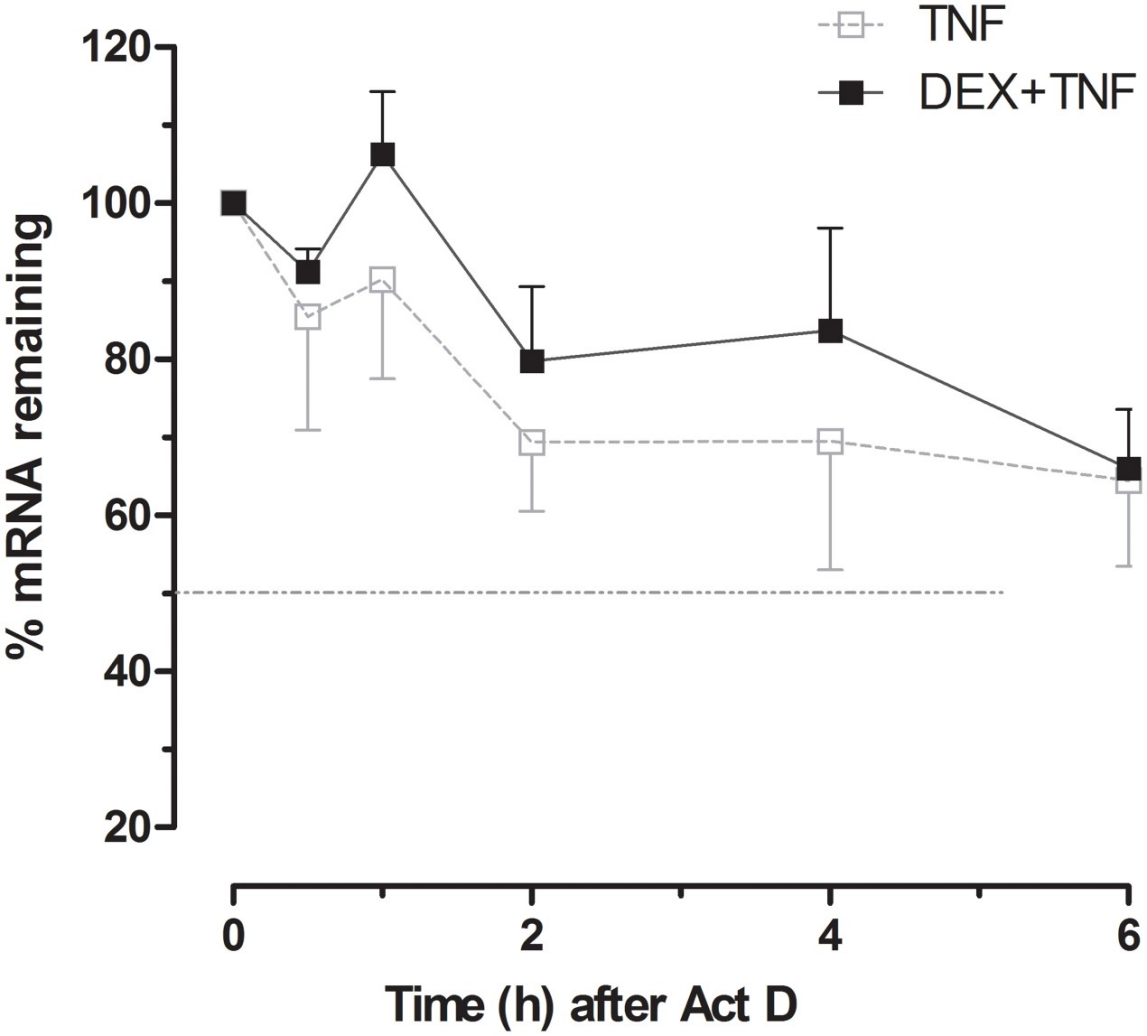
Effects of DEX on PTX3 mRNA stability. Growth-arrested HASMC were treated with TNF (10ng/ml) in the absence or presence of DEX (10^−6^ M) for 12 h. Cells were then washed and incubated with Act D (5 μg/ml). Total RNA was extracted and quantified by real-time PCR. Results are expressed as % mRNA remaining over time after Act D treatment from 3 different primary HASM cell lines isolated from 3 different donors.

Since DEX did inhibit TNF-induced PTX3 promoter activity while augmenting TNF-induced PTX3 secretion, we next examined whether DEX affects TNF-induced PTX3 secretion via the modulation of its mRNA stability. HASMC were treated with TNF, in the absence or presence of DEX, for 9 h. Cells were then washed and incubated with Act D. Total RNA was extracted following 0, 0.5, 1, 2, 4 and 6 h incubation with Act D, and PTX3 mRNA expression was quantified by real-time RT-PCR. As shown in Fig 5, the mean half-life of the PTX3 mRNA transcript induced by TNF was 0.4 h. The addition of DEX significantly increases PTX3 mRNA stability by 3 fold when compared to TNF alone. This effect corroborates by the decay kinetics, where DEX significantly slows the decay rate of PTX3 when added to TNF (0.49 ± 0.13 vs 1.7 ± 1.5). These results indicate that a post-transcriptional mechanism mediates DEX effect on TNF-induced PTX3 secretion.

### DEX induces PTX3 via p42/p44 ERK-MAPK pathways

**Fig 6:**
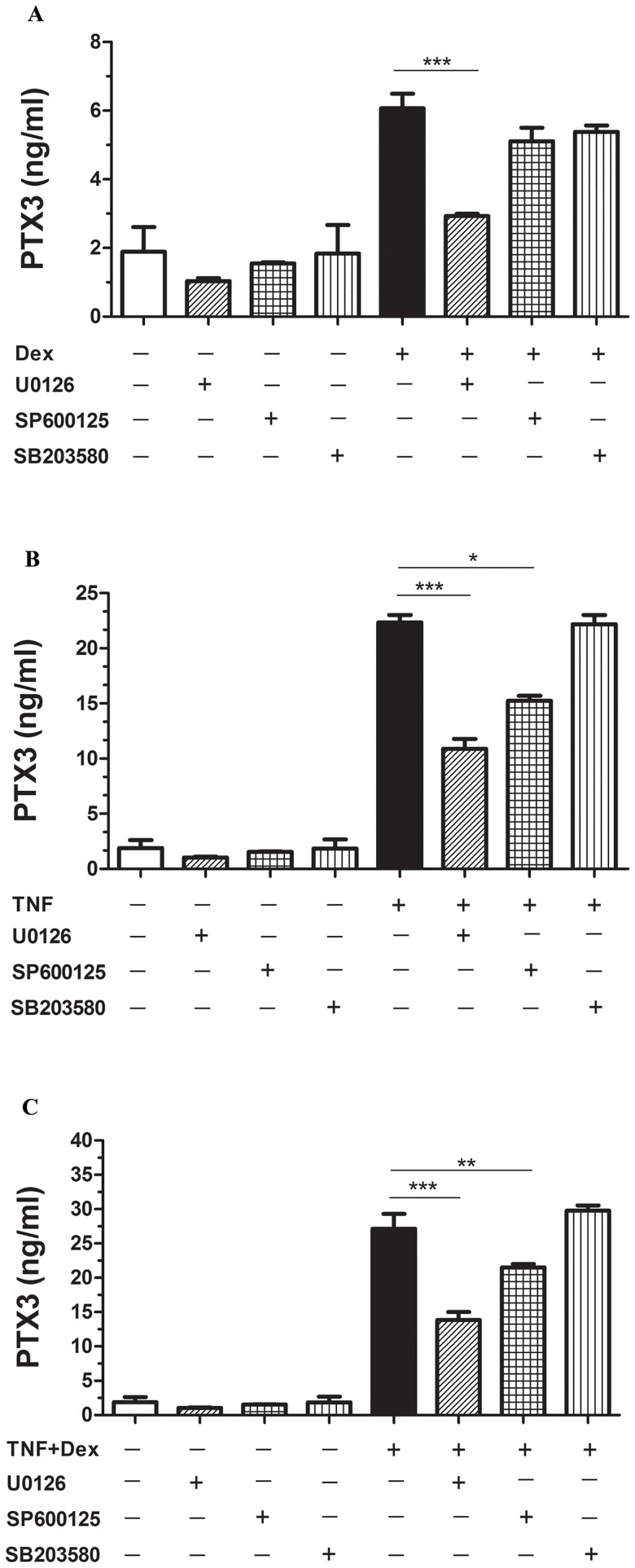
p42/p44 ERK MAPK inhibitors abrogate DEX mediated PTX3 release from HASMC. Growth-arrested cells were left unstimulated (medium alone), treated with DEX (1μM), TNF alone or combination, and with inhibitors of p38 MAPK (SB-203580, 10μM), p42/p44 ERK (U-0126, 10μM), JNK (SP-600125, 50nM) added 30 min before. Results are representative means ±SD of triplicate values from 3 different donors. *P<0.05, **P<0.01***P<0.001, compared with DEX, TNF alone or DEX combined TNF stimulated cells.

We have previously shown TNF-dependent increase of PTX3 is mediated through JNK and ERK1/2 MAPK pathways [15], Whether DEX operates through the same mechanism is not known. PTX3 protein secretion was evaluated in growth-arrested HASMC were either left unstimulated (medium alone) or treated with DEX (10^−6^ M) for 24 h in the presence or absence of the inhibitors of JNK (SP600125 50nM), p42/p44 ERK (U-0126, 10μM) and p38 MAPK (SB-203580, 10μM) added 30 min before. We found that cell treatment with ERK1/2 inhibitor U-0126, but not the p38 or JNK inhibitors, significantly inhibited the ability of DEX (P<0.001, n = 3, Fig 6A) and TNF (Fig 6B) either alone or in combination (Fig 6C) to increase in PTX3 secretion. Interestingly, DEX alone has no effect of ERK1/2 phosphorylation as previously showed [18,19]. However, DEX inhibited significantly TNF mediated ERK1/2 phosphorylation (S2 Fig). Also, HASMC treatment with JNK inhibitor SP600125 partially inhibited TNF-induced PTX3 secretion (Fig 6B) [17]. These data suggest that DEX affect TNF-dependent secretion of PTX3 via p42/p44 ERK-MAPK pathway in HASMC.

## Discussion

Several studies demonstrated that in addition to its contractile properties, ASM can potentially contribute to the pathogenesis of asthma by secreting cytokines and inflammatory mediators, which in turn lead to the activation of crucial tissue remodeling cascade in the airways [11, 20–25]. Previously we showed enhanced PTX3 expression in the lungs of subjects with severe allergic asthma compared to healthy donors [26]. In particular, ASM cells were the main producers of PTX3 *in vivo* and upon proinflammatory cytokine stimulation *in vitro* [9, 17]. In the present study, we aimed at clarifying the effect of GC on PTX3 in HASMCs and in the context of TNF stimulation. We demonstrated that DEX alone induced PTX3 expression at mRNA and protein level in HASMC but has no significant effect on driving PTX3 promoter activity. DEX combined with TNF resulted in enhanced PTX3 protein release in HASMC. Pharmacological inhibition of ERK1/2 but not JNK1/2 or P38 MAPK significantly decreased the effect of DEX either alone or in combination with TNF on PTX3 protein release. Also, DEX inhibited TNF mediated PTX3 promoter activity in HASMC transiently transfected with a 1.1 Kb PTX3 proximal promoter construct [17]. Further, DEX increased TNF induced PTX3 mRNA stability that may account for enhanced protein expression and release. Taken together, our data suggest that DEX regulates PTX3 expression in HASMC by at least post-transcriptional (i.e. mRNA stability) mechanisms and may involve ERK1/2 pathway.

Emerging evidence suggests the involvement of PTX3 as a structural component of ECM [27–29]. In particular, during ovulation, PTX3 is synthesized by granulosa cells in response to hormone stimulation and deposited in the cumulus oophorus matrix [27]. PTX3 was shown to play an essential role in the organization of ECM by cross-linking of hyaluronan (HA) via interaction with TNF-stimulated gene 6 (TSG6) and Inter-α-trypsin inhibitor (IαI) [28]. These studies suggest that PTX3 is a component of ECM essential for HA organization and raises the possibility of a similar localization and function of this molecule in certain HA enriched inflammatory tissues [30]. In concert with this suggestion, we recently demonstrated that PTX3 deficiency resulted in excessive inflammation, AHR, and airway remodeling using OVA model of asthma [10]. *PTX3*^*-/-*^ mice exhibit a Th17 dominant inflammatory response as compared to *PTX3*^*+/+*^ mice in response to OVA. Collectively these findings suggest that PTX3 is part of the critical protective mechanisms by regulating the functional capacity of immune and structural cells. It is also tempting to speculate that PTX3 released from HASMC upon GCs stimulation prevents the excess of inflammatory cells recruitment and airway remodeling, hence identifying a new mechanism by which GC mitigate airway inflammation in asthma. In support of this suggestion, we previously showed that PTX3 differentially modulates the expression of inflammatory genes in ASM cells. Indeed, while we previously showed that PTX3 had no effect on the expression of TGF-beta, IL-6 [9], IL-8 or IL-17A (data not shown), PTX3 was able to significantly induce eotaxin1/CCL11 secretion [9]. Eotaxin-1 /CCL11, besides its role in attracting eosinophils, can exert a specific regulatory function of neutrophil recruitment *in vivo* [31] through the down-regulation of CXC chemokine CXCL8/IL-8 [32]. Hence, it is plausible that PTX3-mediated eotaxin-1/CCL11 release may down-regulate exaggerated neutrophilic inflammation, via the suppression of CXC chemokine, especially in the context of severe asthma. In accordance with this possibility, previous reports showed that PTX3 dampens neutrophil recruitment *in vivo* via a P-selectin dependent mechanism [33]. Also, PTX3 deficient mice develop more myocardial damage associated with more neutrophil infiltration in a model of cardiac ischemia-reperfusion injury [34]. Future studies are needed to investigate the mechanisms underlying the gene-specific effects of PTX3 on the expression of inflammatory genes.

Airway remodeling is a key feature of asthma and is believed to contribute to the decline in lung function observed during chronic disease. Structural changes in the asthmatic bronchial wall include epithelial shedding, deposition of extracellular matrix (ECM) proteins, mucus gland hyperplasia, subepithelial fibrosis, enhanced thickness of smooth muscle layer, and angiogenesis [35, 36]. It is a generally held belief that continued cycles of inflammation driven by allergen exposure ultimately lead to the development of remodeling. However, it has been proposed that inflammation and remodeling may occur in parallel rather than sequentially and that the interaction of the immune system with the lung structural cells is critical in both initiation and development of chronic disease [37]. Besides the role of PTX3 effect in inflammation [10], we previously showed that PTX3 inhibits FGF2-induced ASM cells migration *in vitro* [9] significantly. Furthermore, GC has also been shown to have a profound inhibitory effect on ASM cells migration [19, 38]. Together, these studies suggest that GC may modulate ASM migration via PTX3, a hypothesis that will be examined *in vivo* in our future studies.

GC exert most of their effect on target cells by directly binding to the cytosolic GR triggering its dimerization and translocation to the nucleus. Once in the nucleus, the GR can bind specific sequences of nucleic acids called GC response elements (GRE) in the promoter region of steroid-target genes. Such a process is called transactivation. Alternatively, the GR can directly bind to and suppress other transcription factors through a process called “transrepression”. GR can also influence cell function by modulating various intracellular signaling pathways in a rapid “non-genomic” or “non-transcriptional” manner [39, 40]. Doni A. and coworkers [7] demonstrated that the regulation of PTX3 by GCs is highly cell-specific, with an inhibition in hematopoietic cells (macrophages and DC) and an induction in non-hematopoietic cells (fibroblasts and endothelial cells) where GR plays a critical role evidenced by inhibitory effect of GR antagonist (RU486) [7]. In the present study, we found that DEX enhances the expression of PTX3 both at the protein and mRNA levels that are GR-dependent. Surprisingly, DEX did not influence PTX3 promoter activity as revealed by the proximal-luciferase reporter plasmid. However, it is essential to highlight that this construct only recapitulates part of the complexity of the human PTX3 promoter. Indeed, two regulatory elements upstream of the PTX3 promoter were recently shown to regulate its expression differently [41]. The first element or enhancer 1 is located 230 kb upstream of the PTX3 promoter and mediates the action of inflammatory transcription factors; whereas enhancer 2 is located 350 Kb upstream of the PTX3 promoter and encompasses PTX3 second exon, is implicated in pre-initiation complex assembly. Whether DEX differentially modulates the PTX3 gene regulation through these enhancers in HASMC needs further investigation.

GCs have been shown to regulate gene expression post-transcriptionally by altering mRNA stability of numerous targets, including growth hormone, fatty acid synthetase, regulated in development and DNA damage-1(REDD1), inflammatory response proteins, collagenase, and cyclin D3 [42–46]. In the present study, DEX increased TNF-induced PTX3 mRNA stability indicating the involvement of post-transcriptional mechanism as has been shown in mononuclear cells upon LPS stimulation in the context of sepsis [47].

It has become clear that DEX through binding to GR can influence cell function by modulating various intracellular signaling pathways in a rapid “non-genomic” or “non-transcriptional” manner [39, 40]. In this study, pharmacological inhibitors of MAPK (ERK1/2) abolished DEX-induced PTX3 production from HASMC, suggesting the involvement of MAPK pathways [48]. Previous studies have shown that DEX does not affect ERK1/2 phosphorylation [18, 19] in ASM cells similar to our data (S2 Fig). This may suggest that the effect of ERK1/2 inhibitor in down regulating PTX3 induced by DEX is mediated through an indirect mechanism that needs to be determined. For instance, eotaxin-1/CCL11 expression can be induced by PTX3 in ASM cells [9]. Interestingly, eotaxin1/CCL11 can induce ERK1/2 activation pathway. Furthermore, PTX3 is a multi-domain protein and may interact directly with many ligands expressed on ASM cells including Fc receptors [49] that are known mediators of ERK1/2 [50] may induce PTX3 in an autocrine manner, hence explaining the effect of ERK1/2 inhibitor in our study. Further study is needed to clarify this effect in HASM cells.

In conclusion, DEX induces PTX3 gene expression in HASMC. DEX alone, or in combination with TNF, is dependent on de novo mRNA synthesis and through GR. The mechanisms of the enhanced PTX3 expression may depend on the post-transcriptional mechanism, in part due to the mRNA stability. DEX via ERK MAPK appeared to be required for the induction of PTX3 synthesis and release in HASMC. Our study suggests a novel mechanism involving PTX3 by which HASMC may regulate airway inflammation and immune response in asthma.

## Supporting information

S1 Fig Actinomycin D (5ug/ml) has a no significant effect on HASMC viability. Cells were growth arrested by FBS deprivation and then stimulated in fresh FBS-free media with a graded concentration of actinomycin D (1, 5 and 10 μg/ml) for 24hrs. Cells were then harvested and analyzed for cell viability using propidium iodide combined with flow cytometry (A) or trypan blue exclusion (B). Data is representative of three cell lines.
(PDF)

S2 Fig DEX inhibits TNF induced ERK1/2 phosphorylation in HASMC. Growth-arrested cells were left unstimulated (medium alone), treated with DEX (1μM), TNF alone or combination for 10 and 20 min then lysed. Results are representative means ±SD of triplicate values from 2 different experiments.
(PDF)
