# Supplementary figures and images for: Glucocorticoids regulate pentraxin-3 expression in human airway smooth muscle cells
Source: PLoS One. 2019 Aug 22;14(8):e0220772. doi: 10.1371/journal.pone.0220772 (PMC6706008; doi:10.1371/journal.pone.0220772)

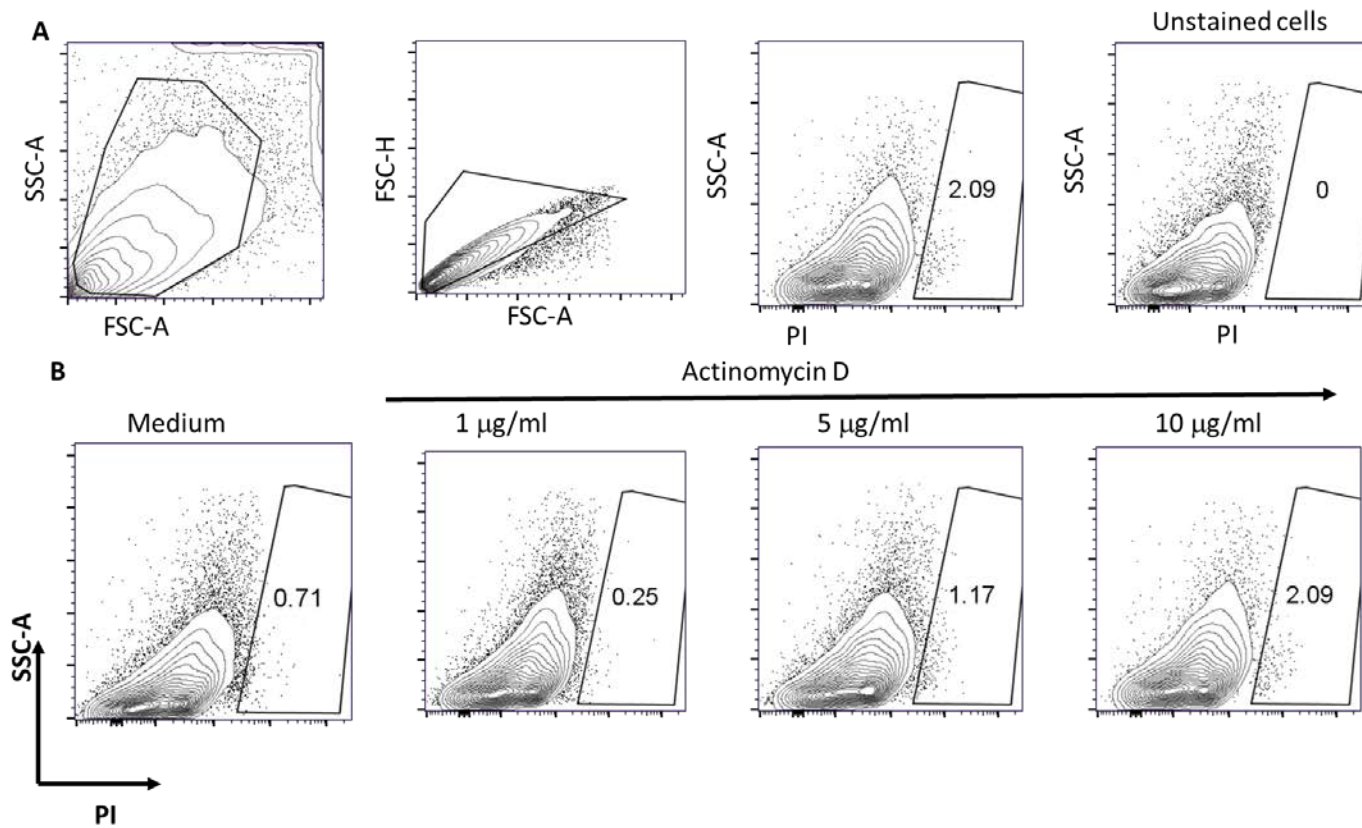

**B** Viability count using trypan blue staining

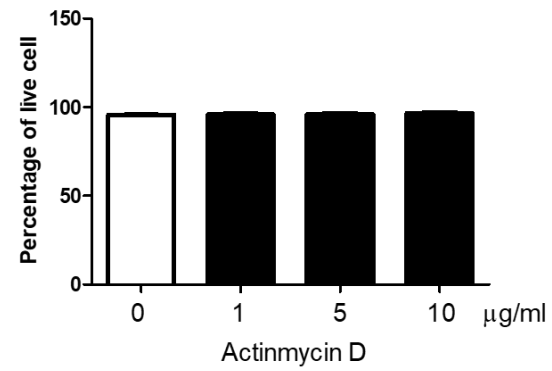

S1 Fig

Supplement: S1 Fig — Cells were growth arrested by FBS deprivation and then stimulated in fresh FBS-free media with a graded concentration of actinomycin D (1, 5 and 10 μg/ml) for 24hrs. Cells were then harvested and analyzed for cell viability using propidium iodide combined with flow cytometry (A) or trypan blue exclusion (B). Data is representative of three cell lines. (PDF) [file pone.0220772.s001.pdf]

**A**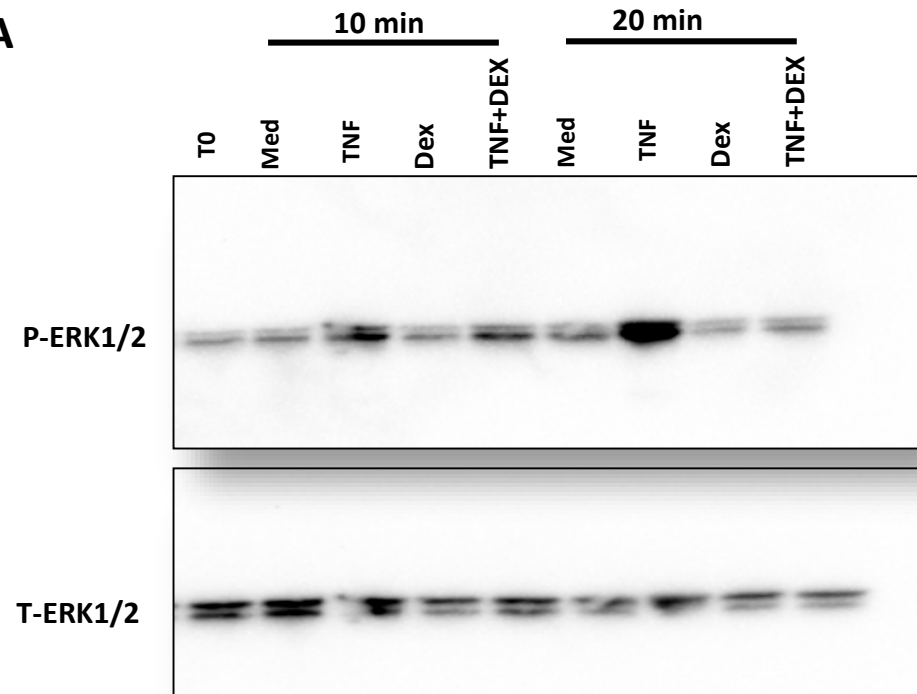**B**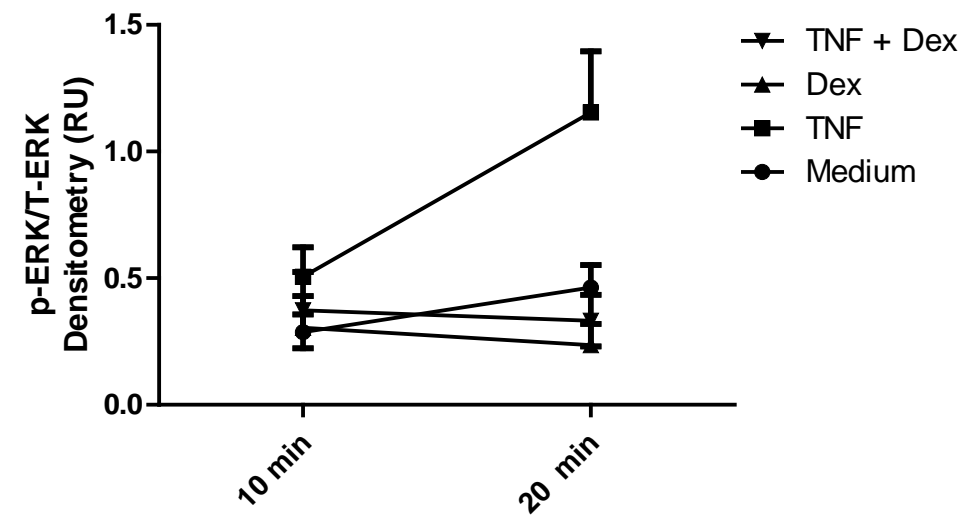

S2 Fig

Supplement: S2 Fig — Growth-arrested cells were left unstimulated (medium alone), treated with DEX (1μM), TNF alone or combination for 10 and 20 min then lysed. Results are representative means ±SD of triplicate values from 2 different experiments. (PDF) [file pone.0220772.s002.pdf]
